# Exploring the occurrence of *Listeria* in biofilms and deciphering the bacterial community in a frozen vegetable producing environment

**DOI:** 10.3389/fmicb.2024.1404002

**Published:** 2024-07-10

**Authors:** Nadja Pracser, Eva M. Voglauer, Sarah Thalguter, Ariane Pietzka, Evelyne Selberherr, Martin Wagner, Kathrin Rychli

**Affiliations:** ^1^FFoQSI GmbH-Austrian Competence Centre for Feed and Food Quality, Safety and Innovation, Tulln, Austria; ^2^Austrian National Reference Laboratory for Listeria monocytogenes, Institute of Medical Microbiology and Hygiene, Austrian Agency for Health and Food Safety, Graz, Austria; ^3^Clinical Department for Farm Animals and Food System Science, Centre for Food Science and Veterinary Public Health, University of Veterinary Medicine Vienna, Vienna, Austria

**Keywords:** microbiota, food processing, colonization, 16S rRNA, microbial interaction

## Abstract

The establishment of *Listeria (L.) monocytogenes* within food processing environments constitutes a significant public health concern. This versatile bacterium demonstrates an exceptional capacity to endure challenging environmental conditions in the food processing environment, where contamination of food products regularly occurs. The diverse repertoire of stress resistance genes, the potential to colonize biofilms, and the support of a co-existing microbiota have been proposed as root causes for the survival of *L. monocytogenes* in food processing environments. In this study, 71 sites were sampled after cleaning and disinfection in a European frozen vegetable processing facility, where *L. monocytogenes* in-house clones persisted for years. *L. monocytogenes* and *L. innocua* were detected by a culture-dependent method at 14 sampling sites, primarily on conveyor belts and associated parts. The presence of biofilms, as determined by the quantification of bacterial load and the analysis of extracellular matrix components (carbohydrates, proteins, extracellular DNA) was confirmed at nine sites (12.7%). In two cases, *L. innocua* was detected in a biofilm. Furthermore, we explored the resident microbial community in the processing environment and on biofilm-positive sites, as well as the co-occurrence of bacterial taxa with *Listeria* by 16S rRNA gene sequencing. *Pseudomonas*, *Acinetobacter*, and *Exiguobacterium* dominated the microbial community of the processing environment. Using differential abundance analysis, amplicon sequence variants (ASVs) assigned to Enterobacterales (*Enterobacter*, *Serratia*, unclassified *Enterobacteriaceae*) and *Carnobacterium* were found to be significantly higher abundant in *Listeria*-positive samples. Several *Pseudomonas* ASVs were less abundant in *Listeria*-positive compared to *Listeria*-negative samples. *Acinetobacter, Pseudomonas*, *Janthinobacterium*, *Brevundimonas*, and *Exiguobacterium* were key players in the microbial community in biofilms, and *Exiguobacterium* and *Janthinobacterium* were more relatively abundant in biofilms. Further, the microbial composition varied between the different areas and the surface materials.

## 1 Introduction

*Listeria (L.) monocytogenes* is of particular concern due to its versatility and ability to adapt to harsh environmental conditions such as low temperatures (Schmid et al., 2009; Muchaamba et al., 2022; Osek et al., 2022). *L. monocytogenes* is further able to persist for years in diverse food processing environments despite hygiene measures (Guidi et al., 2021; Chen et al., 2022; Sullivan et al., 2022; Chowdhury and Anand, 2023; Schiavano et al., 2023) elevating the risk of food contamination. Long-term survival of *L. monocytogenes* has been previously linked to gene content involved in stress resistance, which provides tolerance against disinfectants, a wide range of temperatures, acidic and alkaline conditions, and high salinity (Bucur et al., 2018; Lakicevic et al., 2022). However, there are indications that the genomic content is not the only factor explaining the persistence of *L. monocytogenes*. Our previous study confirmed the presence of in-house clones in a European frozen vegetable-producing environment. However, no differences in the stress resistance gene pattern were observed between in-house and non-in-house clones (Pracser et al., 2024). Other factors that could support the persistence of *L. monocytogenes* in food processing environments include biofilms and interactions with co-occurring microbiota (Møretrø and Langsrud, 2017; Tan et al., 2019; Finn et al., 2023).

Biofilms are composed of a multitude of bacterial cells embedded in a self-produced extracellular matrix consisting of carbohydrates, proteins and extracellular DNA (eDNA). The biofilm architecture allows for the exchange of genetic material, provides nutrients and protects cells from stressors such as disinfectants or desiccation (Esbelin et al., 2018; Li et al., 2021; Flemming et al., 2023). Various studies have investigated the biofilm-forming potential of *L. monocytogenes* and other bacterial species commonly found in food processing environments in single- and multispecies biofilms in the lab. Indeed, there are extensive variations in the biofilm-forming ability of different bacterial species and strains (e.g., Wagner et al., 2021; Di Ciccio et al., 2022). Multispecies biofilm models showed that *L. monocytogenes* is able to colonize a monospecies *Pseudomonas* biofilm (Rodríguez-López et al., 2022; Sterniša et al., 2023) or even a multi-taxa biofilm (Rolon et al., 2024b). These studies showed that the bacterial interactions in multi-species biofilms are complex and that *L. monocytogenes* within biofilms is protected from environmental stressors, e.g., from antimicrobials. For example, the biofilm matrix reduces the diffusion of antimicrobials or extracellular enzymes in the matrix degrade antimicrobials, resulting in lower concentration of antimicrobials (Maillard and Centeleghe, 2023). A shortcoming of these studies is that the behavior of *Listeria* within a biofilm has only been studied in biofilm models.

The composition of the resident microbiota of a food producing environment is influenced by food products and diverse environmental factors like nutrient avaiablility, humidity, sanitation methods or temperature, and also by the workers. Therefore, the microbiota of food processing environments is often composed of versatile microorganisms, which can adapt to various environmental stressors (Maes et al., 2019). *Pseudomonas*, *Acinetobacter*, *Staphylococcus*, *Psychrobacter*, *Stenotrophomonas*, *Serratia*, and *Microbacterium* are prevalent in food processing environments. In addition, the environmental conditions may promote the growth and increase the relative abundance of certain bacterial taxa. The resident microbiota may interact competitive or cooperative with other bacteria including food pathogens (De Filippis et al., 2021; Fagerlund et al., 2021; Tadielo et al., 2023; Xu et al., 2023). Diverse bacterial taxa isolated from food processing environments were shown to influence the survival and growth of *Listeria* under laboratory conditions (Sinclair et al., 2022; Lake et al., 2023; Rolon et al., 2024a,b). Positive and negative associations of members of microbial communities with *L. monocytogenes* and other *Listeria* spp. were already investigated in meat processing facilities (Zwirzitz et al., 2021; Belk et al., 2022; Cherifi et al., 2022), tree fruit packing facilities (Rolon et al., 2023), the fish, meat and dairy industry (Rodríguez-López et al., 2019), and distribution centers for fresh produce (Townsend et al., 2023). However, there is still limited knowledge on the extent to which *Listeria* spp. are present in biofilms in the food-producing environment and which bacterial taxa are present in biofilms.

In this study, we examined the presence of *Listeria* spp., the presence of biofilms, and the composition of the microbial community in a European frozen vegetable processing facility. Sampling was performed at 71 environmental sites, including food-contact surfaces and non-food-contact surfaces in three different subareas. To our knowledge, this is the first study describing the presence of *Listeria* in biofilms and the specific residing microbiota in a frozen vegetable processing environment.

## 2 Materials and methods

### 2.1 Sampling

Sampling of environmental surfaces was performed on two sampling visits in April and May 2022 in a European frozen vegetable processing facility. On the first visit, samples were taken in the subarea “Production” where processing lines for blanching and deep-freezing of fresh vegetables were located. The second sampling visit comprised two subareas. In “Packaging Room A,” processing lines for mixing vegetables and packaging of frozen vegetables were sampled. In “Packaging Room B,” sampling was conducted on processing lines for frozen fried vegetable products and the packaging of frozen vegetable products. All samples were taken after cleaning and disinfection and included food-contact, indirect food-contact (surfaces touching, e.g., parts of equipment, which are in direct food contact) and non-food-contact surfaces. In total, 71 regular samples, seven supplementary samples and 19 negative controls were taken (Supplementary Tables S1, S2).

Each site was sampled using two sets of sampling devices: First, for biofilm detection a scraper (Cell Scraper; length: 225 mm, blade width: 20 mm; Carl Roth) and Nylon^®^ flocked swabs (552C, FLOQSwabs, COPAN) (Maes et al., 2017; Wagner et al., 2020) were used. The sampling surface areas were horizontally and vertically wiped with the scraper and flocked swabs. Second, for *Listeria* detection and analysis of the microbial community, a larger area of the same sampling site was sampled with a hydrated polyurethane sponge (PU) swab in foil bags (HiCap Neutralizing swabs, Nasco Whirlpak). The size of surface areas of sites sampled for biofilm detection ranged from 5 to 300 cm^2^ and for *Listeria* detection and microbial community description from 20 to 1,000 cm^2^. Surface areas that were not accessible with both sets of devices (e.g., too small for PU swabs such as the inner wall of a water hose outlet), were only sampled with scraper and flocked swabs. Scraper and flocked swabs were transported in 10 mL of ¼ Ringer solution (B. Braun Austria GmbH). All samples were transported under cool conditions and were processed within 24 h.

### 2.2 Culture-depended detection of *Listeria*

*Listeria* spp. were recovered from surfaces wiped with PU-swabs. To each foil bag containing a PU-swab, 10 mL of 1X Phosphate Buffered Saline (PBS) (Thermo Fisher Scientific) were added. The samples were homogenized using a Stomacher blender at middle speed for 1 min. From the homogenized suspension, 8 mL were removed from the foil bag and transferred to a separate tube for total DNA recovery (see Section 2.3). Isolation of *Listeria* was performed according to ISO 11290-1:2017 with modifications. 50 mL of half-Fraser broth (Noack) were added to each foil bag containing a PU-swab. The samples were incubated at 30°C for 36 h for primary enrichment. For the secondary enrichment, 100 μL of the primary enrichment were transferred to 9 mL of Fraser broth (Noack) and incubated at 37°C for 48 h. The primary and secondary enrichment were plated out on ALOA (Merck Millipore) and PALCAM (Biokar Diagnostics) agar plates. The agar plates incubated at 37°C for 48 h.

### 2.3 DNA extraction

Total DNA was extracted from putative *Listeria* spp. colonies on the selective agar plates using a Chelex-based (Chelex 100, Sigma-Aldrich) extraction procedure (Walsh et al., 1991). A Chelex 100 stock solution was prepared with 2.5 g Chelex 100, 2.5 mL 0.01 M Tris–HCl (pH 7.0) and 95 mL autoclaved distilled water. Putative *Listeria* isolates were transferred from selective agar plates to 1.5 mL tubes containing 100 μL 0.01 M Tris–HCl (pH 7.0) and 400 μL of the Chelex stock solution, respectively. The suspensions were vortexed and incubated at 95°C for 10 min shaking (500 rpm). After centrifugation at 15,000 × g for 30 s, 150 μL of the supernatant containing extracted DNA of each *Listeria* isolate was transferred to a new 1.5 mL tube.

In addition, bacterial cells were recovered from samples wiped with PU-swabs and scraper and flocked swabs, respectively, for subsequent DNA extraction. The 8 mL bacterial cell suspension recovered from sampling with PU-swabs (see Section 2.2) was centrifuged at 3,220 × g for 10 min at 20°C. The resulting pellet was resuspended in 500 μL PBS (Thermo Fisher Scientific) and stored at −80°C until DNA extraction.

The sample processing of scraper and flocked swabs was done according to a previous study (Wagner et al., 2020). Briefly, we added 2 g of hydrated cation exchange resin (CER, Amberlite^®^ HPR110, 20–50 mesh, Sigma-Aldrich) to each sample collected with scraper and flocked swabs, followed by 15 min of shaking at 500 rpm. Then, the suspension was centrifuged at 3,220 × g at 20°C for 15 min. The supernatant was sterile filtered through a 0.22 μm filter membrane (Filtropur S0.2, Sarstedt AG & Co KG) and stored at −20°C until analysis of extracellular matrix components (see Section 2.5). The cell pellet including CER was stored at −20°C until DNA extraction. Bacterial cell pellets including CER were thawed to prepare for DNA extraction at room temperature and 5 mL 1X PBS (Thermo Fisher Scientific) were added to each sample. Mixing of the samples was achieved via vortex agitation. Subsequently, CER was allowed to settle for 2 min resulting in a separation of the supernatant and CER. The supernatant was transferred to a new centrifugation tube. The washing step with PBS was repeated twice. Then, the collected supernatant was centrifuged for 5 min at 3,220 × g. The supernatant was discarded resulting in the recovery of a bacterial cell pellet without CER.

Total DNA was extracted from bacterial cell pellets using the DNeasy^®^PowerSoil^®^ProKit (Qiagen) according to manufacturer’s instructions with small modifications. For bacterial cell disruption, bead beating (4.5 m/s, 45 s, 5 repetitions) with Matrix Lysis A (MP Biomedicals) was performed on a FastPrep-24^™^ 5G (MP Biomedicals). The elution step was performed twice with 25 μL 70°C sterile water (Sigma-Aldrich). DNA concentration was determined using the Qubit fluorometer (Invitrogen) with the Qubit high sensitivity dsDNA kit (Invitrogen) and 2 μL of DNA. DNA was stored at −80°C until further analysis.

In addition, *L. monocytogenes* isolates were sent to the Austrian National Reference Laboratory for *Listeria monocytogenes* (AGES) for total DNA extraction using the MagAttract high-molecular-weight (HMW) DNA kit (Qiagen). Total DNA of *L. innocua* isolates was extracted with the GeneJet Genomic DNA Purification kit (Thermo Fisher Scientific) following the manufacturer’s instructions for purification of genomic DNA from gram-positive bacteria with minor modifications. The final elution step was carried out with 50 μL 37°C sterile water (Sigma-Aldrich).

### 2.4 Molecular characterization

For differentiation of *Listeria* spp., two PCRs were performed (Border et al., 1990; Bubert et al., 1999). One PCR targeted the *hly* gene, which encodes for listeriolysin O (primer sequences: 5′-CCT AAG ACG CCA ATC GAA-3′, 5′-AAG CAC TTG CAA CTG CTC-3′) for identification of *L. monocytogenes*, and the fragment of 16S rRNA for detection of *Listeria* spp. (primer sequences: 5′-CAG CAG CCG CGG TAA TAC-3′, 5′-CTC CAT AAA GGT GAC CCT-3′). The reaction volume of the PCR was 25 μL and contained 1.5 μL of each primer (stock concentration 18 μM, final concentration: 1.08 μM), 8.5 μL of sterile water (Sigma-Aldrich), 2.5 μL of 10X PCR Buffer (-MgCl_2_) (Invitrogen), 0.75 μL 50 mM MgCl_2_, 5 μL of 4 mM deoxynucleotide triphosphate mix (dNTPs, Thermo Fisher Scientific), 0.25 μL of 5 U/μl Platinum™ Taq DNA Polymerase (Invitrogen), and 2 μL of DNA template. PCR was carried out with following settings: initial denaturation at 94°C for 2 min, 30 cycles of denaturation at 94°C for 30 s, annealing at 50°C for 30 s, extension at 72°C for 1 min, followed by a final extension step at 72°C for 5 min.

The second multiplex PCR approach targeted the *iap* gene for differentiation of *Listeria* spp.. Primers specific for *L. monocytogenes* (5′-CAA ACT GCT AAC ACA GCT ACT-3′), *L. innocua* (5′-ACT AGC ACT CCA GTT AAA C-3′), *L. grayi* (5′-CCA GCA GTT TCT AAA CCT GCT-3′), the *L. ivanovii/L. seeligeri/L. welshimeri* group (5′-TAA CTG AGG TAG CGA GCG AA-3′), were used in pair with the primer sequence 5′-TTA TAC GCG ACC GAA GCC AAC-3′. The reaction volume of the PCR was 25 μL and contained 2 μL of each primer (stock concentration 1.6 μM, final concentration: 128 nM), 4.45 μL of sterile water (Sigma-Aldrich), 2.5 μL of 10X PCR Buffer (-MgCl_2_) (Invitrogen), 0.75 μL 50 mM MgCl_2_, 5 μL of 4 mM deoxynucleotide triphosphate mix (dNTPs, Thermo Fisher Scientific), 0.3 μL of 5 U/μl Platinum^™^ Taq DNA Polymerase (Invitrogen), and 2 μL of DNA template. PCR was carried out with following settings: initial denaturation at 94°C for 2 min, 30 cycles of denaturation at 94°C for 30 s, annealing at 56°C for 30 s, extension at 72°C for 30 s, followed by a final extension step at 72°C for 5 min. PCR products were visualized by gel electrophoresis.

A quantitative real-time PCR (qPCR) targeting the 16S rRNA gene (primer sequences: 5′-CCT ACG GGA GGC AGC AG-3′, and 5′-ATT ACC GCG GCT GCT GG-3′) was applied to calculate total bacterial cell equivalents (BCE) in all samples as previously described (Dixon et al., 2019; Wagner et al., 2020). Briefly, the volume of a single qPCR reaction was 20 μL containing 1 μL template DNA, 7 μL sterile water (Sigma-Aldrich), 1 μL of each primer (final concentration 250 nM), and 10 μL Brilliant III Ultra-Fast SYBR^®^ Green qPCR master mix with low ROX (Agilent). Amplification was performed with one cycle at 95°C for 3 min, 40 cycles at 95°C for 5 s and at 60°C for 20 s, followed by generation of a melting curve at 95°C for 1 min, 60°C for 30 s, and 95°C for 30 s. Copy numbers of background controls of the DNA extraction kit, which determined the bacterial contamination of reagents, were subtracted from copy numbers of the samples. An average of 5.3 16S rRNA gene copy numbers was estimated using rrnDB for calculation of total BCE (Větrovský and Baldrian, 2013; Stoddard et al., 2015).

Whole genome sequencing of *L. monocytogenes* isolates was carried out by AGES. Briefly, whole genome sequencing libraries were prepared using the Nextera XT kit (Illumina) and paired-end sequencing (2 × 300 bp) was performed on a MiSeq platform (Illumina). Whole genome sequencing of *L. innocua* isolates was performed by Microsynth (Balgach, Switzerland). Briefly, Illumina Nextera two-step PCR libraries were prepared and paired-end sequencing on an Illumina MiSeq platform (2 × 250 bp) was done.

DNA from samples taken with PU-swabs was sent to Microsynth (Balgach, Switzerland) for sequencing of the V3-V4 region of the 16S rRNA gene. Preparation of Illumina Nextera two-step PCR libraries (Illumina) and paired-end sequencing on an Illumina MiSeq platform (2 × 250 bp) was performed by Microsynth.

### 2.5 Biochemical characterization of biofilm matrix components

The amounts of carbohydrates, eDNA and proteins were determined in the supernatant of the processed biofilm samples.

#### 2.5.1 Presence of carbohydrates in the biofilm matrix

Concentration of carbohydrates in each sample was attained via evaporation for 1 h at 90°C shaking at 300 rpm. A phenol-sulfuric acid method was applied to determine the carbohydrate content in each sample (Masuko et al., 2005). Glucose was used for creating a standard curve that was used for calculation of glucose equivalents in each sample. The limit of quantification (LOQ) was 12.6 mg/L glucose equivalents.

#### 2.5.2 Presence of eDNA in the biofilm matrix

Ethanol precipitation according to Zetzmann et al. (2015) was done in order to precipitate eDNA. To each sample, 0.1 × (of sample volume) 3 M Na-acetate, 0.1 × 0.1 M MgCl_2_ and 2.5 × ice-cold ethanol absolute were added, and samples incubated at −20°C for 24 h. Precipitated DNA was recovered by centrifugation at 20,817 × g for 15 min. The supernatant was discarded, and the pellet was washed with 1 × (of sample volume) 70% ethanol. The centrifugation step was repeated, and the resulting pellet was resuspended in 30 μL of water. Samples were measured for eDNA content on a DeNovix DS-11 FX+ spectrophotometer. The limit of blank (LOB), as assessed from negative precipitation controls, was 2 ng/μl.

#### 2.5.3 Presence of proteins in the biofilm matrix

Precipitation of proteins was done with 0.1 × (of sample volume) TCA/Acetone (1 g/mL) and 0.01 × 2% sodium deoxycholate at 4°C over-night (Rychli et al., 2016). Precipitated proteins were recovered by centrifugation at 20,817 × g at 4°C for 30 min. The pellet was washed with ice-cold acetone and the centrifugation step was repeated. The air-dried pellet was dissolved in 20 μL of 0.05 M Tris–HCl, and the dissolved samples incubated for 3 h at room-temperature. Presence of proteins was determined using the Quant-iT protein assay kit (Invitrogen) according to manufacturer’s instructions. The Quant-iT protein buffer was spiked with bovine serum albumin (final concentration: 1.7 ng/μl BSA), since the sensitivity of the protein assay kit was not high enough for the low biomass samples of the current study. Fluorescence of the spike solution was measured and subtracted from other samples. A limit of blank (LOB) (305 ng) was calculated from the spiked Quant-iT protein buffer solution.

### 2.6 Bioinformatics

#### 2.6.1 Analysis of *Listeria* genome data

Quality of demultiplexed raw reads of all *Listeria* spp. isolates was assessed with fastqc v0.11.9 (Andrews, 2010) and MultiQC v1.0 (Ewels et al., 2016). Read trimming and adapter removal was done with trimmomatic v0.39 (Bolger et al., 2014). Subsequently, adapter-removed and trimmed reads were assembled using SPAdes v3.15.4 (Bankevich et al., 2012) with default settings. Quality of genomes was assessed with QUAST v5.0.2 (Gurevich et al., 2013) and reads were mapped back to the genome assemblies using BBmap v39.01 (Bushnell, 2014) to determine coverage. Subtyping of *L. monocytogenes* isolates was performed using SeqSphere+ v9.0.3 (Ridom GmbH) by the identification of the multi locus sequence typing-sequence type (MLST-ST) and the core genome multi locus sequence typing-complex type (cgMLST-CT) according to the subtyping scheme from Ruppitsch et al. (2015). The MLST-ST of *L. innocua* isolates was determined using the BIGSdb-Pasteur database (Institut Pasteur, France; https://bigsdb.pasteur.fr/). Further, a whole genome SNP analysis of *L. innocua* and *L. monocytogenes* isolates was performed using the CFSAN SNP pipeline v2.2.1 (Davis et al., 2015) with default settings. As reference genome, an internal draft genome was selected based on total length, low number of contigs and coverage. A phylogenetic tree was constructed with the filtered SNP matrix with IQ-TREE v2.0.3 (Minh et al., 2020) using the GTR + ASC substitution model with 1,000 ultrafast bootstrap replicates (Hoang et al., 2017). Visualization of phylogenetic trees was performed with iTOL (Interactive Tree of Life) (Letunic and Bork, 2021).

#### 2.6.2 Analysis of 16S rRNA sequencing data

Analysis of the microbial community with sequencing results from the V3-V4 region of the 16S rRNA gene was carried out as follows: Demultiplexed raw reads were quality checked with fastqc v0.11.9 (Andrews, 2010) and MultiQC v1.0 (Ewels et al., 2016). Removal of residual adapter sequences was done using trimmomatic v0.39 (Bolger et al., 2014). Subsequently, reads were imported into QIIME-2 v2023.2.0 (Bolyen et al., 2019). Reads were trimmed and quality-filtered using dada2 implemented in QIIME-2 and the resulting amplicon sequence variants (ASVs) were taxonomically classified using the SILVA database SSU 138 (Quast et al., 2013). Potential contaminant ASVs were filtered from the ASV table by using the “isNotContaminant” function (threshold 0.5) in the *decontam* v1.20.0 R package for low biomass samples, which applies a prevalence-based method to identify potential contaminants (Davis et al., 2018). Initial dataset exploration was done in R v4.3.1 (R Core Team, 2023) using the *phyloseq* package v1.44.0 (McMurdie and Holmes, 2013). Samples with less than 1,000 total reads were removed from the dataset (*n* = 19). ASVs detected in less than 10% of samples were removed for differential abundance analysis (*n* = 1,026), which was subsequently conducted with *DESeq2* v1.40.2 (Love et al., 2014). For exploring alpha- and beta-diversity, the dataset was rarefied to the lowest read depth (1,280 reads). Alpha diversity was investigated using *vegan* v2.6–4 (Oksanen et al., 2022) with indices Observed, Chao1, Shannon, Simpson, InvSimpson, ACE, and Fisher’s alpha. Beta-diversity was explored using the Bray-Curtis index with a t-distributed stochastic neighbor embedding method [tSNE; *tsnemicrobiota* package v0.1.0 (Lindstrom, 2023)]. Figures were created with *ggplot2* v3.4.4 (Wickham, 2016).

### 2.7 Statistical analyses

The Wilcoxon rank sum test with Benjamini-Hochberg adjustment for *p*-value was used for pairwise comparisons of the alpha-diversity indices Observed, Chao1, Shannon, Simpson, InvSimpson, ACE, and Fisher’s alpha. Differences in the bacterial community compositions (beta diversity) were assessed by calculating a permutational analysis of variance (PERMANOVA) with 5,000 permutations using the “adonis2” function in the *vegan* v2.6–4 package with R v4.3.1. A *p*-value < 0.05 was considered as significant for statistical calculations.

## 3 Results

### 3.1 Bacterial load in a frozen vegetable processing facility

Three different rooms (“Production”, “Packaging Room A”, “Packaging Room B”) were sampled with two sets of sampling devices (scraper and flocked swabs for biofilm detection; PU-swabs for *Listeria* detection and analysis of the bacterial community). The different sampling devices were necessary as the material of the PU sponge swab interferes with biofilm matrix analyses. Scraper and flocked swabs failed to recover *Listeria* spp. and to recover enough biomass for the microbiota analysis. Therefore, sampling sites were additionally wiped with PU sponge swabs.

**Figure 1:**
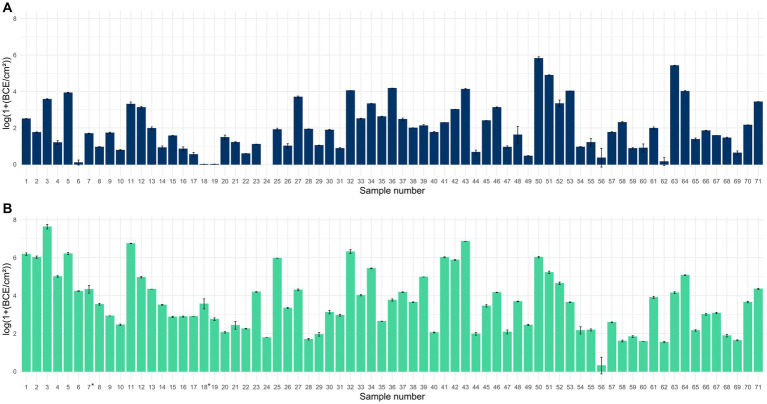
Bar chart displaying the mean bacterial load in log(1+ BCE/cm^2^) ± SD for samples collected with scraper and flocked swabs to detect biofilms **(A)** and for samples collected with PU-swabs **(B)** for microbiota analysis (the addition of 1 to raw BCE/cm^2^ values before log transformation was applied, to only obtain positive values after log transformation, since BCE/cm^2^ values <1 would result in negative values). *: sites 7 and 18 were sampled using two swabs each. Mean values from two different swabs taken at sites 7 and 18, respectively.

Quantification of the 16S rRNA gene revealed the presence of bacteria in all 71 samples collected with PU-swabs (Figure 1; Supplementary Table S1). The highest bacterial load was detected on “blancher 1—conveyor belt—blanching zone” in the “Production” room (site 3) with 7.6 log BCE/cm^2^, on a conveyor belt guide roller in “Packaging Room A” (site 43) with 6.9 log BCE/cm^2^, and in the drain next to blancher in the “Production” room (site 11) with 6.8 log BCE/cm^2^. The lowest BCE was observed on a screw conveyor (site 56) with 0.3 log BCE/cm^2^.

We additionally analyzed the bacterial load in samples collected with scraper and flocked swabs, which are suitable for biofilm analysis. This was essential as the presence of bacteria is a prerequisite for the formation of bacterial biofilms. We detected bacteria in 70 samples ranging from 0.006 to 5.8 log BCE/cm^2^. By trend, the bacterial load was overall higher in samples collected with PU-swabs than in samples collected with scraper and flocked swabs.

### 3.2 Presence of biofilms

**Figure 2:**
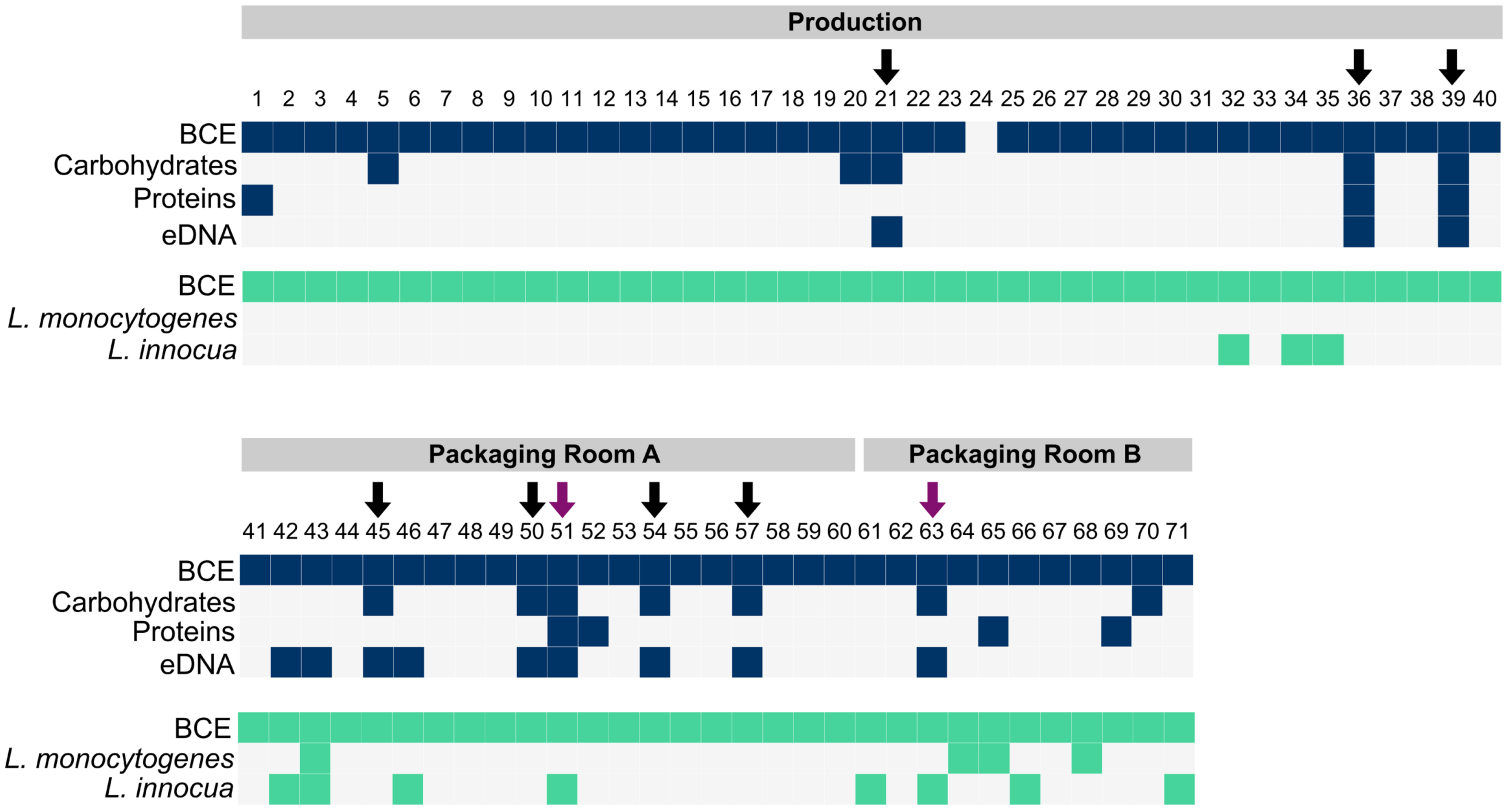
The presence of bacteria (BCE), carbohydrates, proteins and eDNA in samples collected with scraper and flocked swabs for biofilm detection (dark blue), and the presence of bacteria (BCE), *L. monocytogenes*, and *L. innocua* in samples collected with PU-swabs (green) in the production and packaging rooms A and B. Arrows indicate the presence of biofilms. Purple arrows indicate the presence of *Listeria* spp. in biofilms.

For the detection of biofilms, the presence of matrix components such as carbohydrates, proteins, and eDNA was determined. Carbohydrates were detected in 12 samples, proteins in 7 samples, and eDNA in 12 samples (Figure 2). The carbohydrate content ranged from 0.70–13.86 μg/cm^2^, the protein amount ranged from 31.67 to 296.47 ng/cm^2^, and the eDNA concentration ranged from 14.31 ng/cm^2^ to 100.99 ng/cm^2^ (Supplementary Figure S1).

In total, nine biofilm sites (12.68% of 71 total samples) were identified based on the criteria of the presence of at least two matrix components and a positive bacterial cell equivalent (BCE) count (Figure 2). In the “Production” room, biofilms were present on a metal tub (site 21), a conveyor belt (site 36) and an air separator (site 39). In “Packaging Room A”, biofilms were identified on a conveyor belt (site 45), a conveyor screw (site 50), a conveyor belt guide roller (site 51), a filling funnel (site 54), and a metal funnel (site 57). In “Packaging Room B”, a biofilm was detected on a conveyor belt guide roller (site 63).

### 3.3 Presence and characterization of *Listeria* spp.

The presence of *Listeria* spp. was assessed in samples collected with PU-swabs. In total, 14 samples were positive for *Listeria* spp. (Figure 2; Table 1; Supplementary Table S3). In the “Production” room, *L. innocua* (ST1481) was present in a drain (site 32) and on two conveyor belts after the freezer exit (site 34 and 35). In “Packaging Room A,” *L. innocua* (ST2347) was detected on a conveyor belt (site 42) and on conveyor belt guide rollers (site 43, 46, and 51). Additionally, *L. monocytogenes* (ST8-CT8851) was recovered from a conveyor belt guide roller (site 43). In “Packaging Room B,” *L. innocua* (ST2347) was isolated from drains (site 61 and 66) and a conveyor belt guide roller (site 63), *L. innocua* (ST1489) was isolated from a drain (site 71), and *L. monocytogenes* (ST224-CT5623) was detected in a drain (site 64), and on two conveyor belts (site 65 and 68). Of note, the *L. innocua* positive sites 51 and 63, both conveyor belt guide rollers in “Packaging Room A and B”, were also positive for biofilms. In addition, a ST224-CT4656 (*L. monocytogenes*) isolate and a ST1481 (*L. innocua*) were recovered from employees’ shoe soles (Supplementary Tables S2, S3).

**Table 1:** *Listeria* spp. isolated from the frozen vegetable processing environment.

Isolate	Species	Isolation source	MLST-ST	cgMLST-CT*
Li-121	*L. innocua*	Site 42—conveyor belt	2,347	NA
Li-122	*L. innocua*	Site 43—conveyor belt guide rollers	2,347	NA
Li-125	*L. innocua*	Site 46—conveyor belt guide rollers	2,347	NA
Li-130	*L. innocua*	Site 51—conveyor belt guide rollers	2,347	NA
Li-142	*L. innocua*	Site 61—drain	2,347	NA
Li-144	*L. innocua*	Site 63—conveyor belt guide rollers	2,347	NA
Li-147	*L. innocua*	Site 66—drain	2,347	NA
Li-153	*L. innocua*	Site 71—drain	1,489	NA
Li-154	*L. innocua*	Site 88.S—employee’s shoe soles	1,481	NA
Li-264	*L. innocua*	Site 32—drain	1,481	NA
Li-272	*L. innocua*	Site 34—conveyor belt	1,481	NA
Li-273	*L. innocua*	Site 35—conveyor belt	1,481	NA
MRL-22-01015	*L. monocytogenes*	Site 43—conveyor belt guide rollers	8	8,851
MRL-22-01016	*L. monocytogenes*	Site 64—drain	224	5,623
MRL-22-01017	*L. monocytogenes*	Site 65—conveyor belt	224	5,623
MRL-22-01018	*L. monocytogenes*	Site 68—conveyor belt	224	5,623
MRL-22-01019	*L. monocytogenes*	Site 88.S—employees’ shoe soles	224	4,656

NA, not applicable; *: subtyping scheme according to Ruppitsch et al. (2015).

By applying whole genome SNP analysis, 1–3 SNPs could be identified among ST224-CT5623 isolates. Additionally, ST224-CT5623 clustered together in a phylogenetic tree (Supplementary Figure S3; Supplementary Table S5). Moreover, ST224-CT5623 isolates were identified in a previous study as in-house clones surviving for years in the frozen vegetable facility, indicating their re-occurrence in processing lines of “Packaging Room B” over time (Pracser et al., 2024).

*L. innocua* isolates within MLST-ST groups were closely related and clustered together in a phylogenetic tree (Supplementary Figure S2), with 0 SNPs identified among isolates of ST1481 and 0–2 SNPs among isolates of ST2347 through whole genome SNP analysis (Supplementary Table S4).

### 3.4 Bacterial communities in the frozen vegetable processing environment

Microbial diversity within samples was estimated using alpha diversity indices (Observed, Chao1, ACE, Shannon, Simpson, Inversed Simpson, and Fisher) for the different sample groups. No significant differences between alpha diversity indices were observed within *Listeria*-positive and -negative sites (Wilcoxon test, Observed: *p* = 0.306, Chao1: *p* = 0.306, ACE: *p* = 0.326, Shannon: *p* = 0.219, Simpson/InvSimpson: *p* = 0.189, Fisher: *p* = 0.306), within biofilm and non-biofilm harboring sites (Wilcoxon test, Observed: *p* = 0.723, Chao1: *p* = 0.627, ACE: *p* = 0.537, Shannon: *p* = 0.969, Simpson/InvSimpson: *p* = 1, Fisher: *p* = 0.723), as well as within samples from different rooms (Kruskal-Wallis test, Observed: *p* = 0.085, Chao1: *p* = 0.119, ACE: *p* = 0.095, Shannon: *p* = 0.143, Simpson/InvSimpson: *p* = 0.333, Fisher: *p* = 0.085) (Supplementary Figures S4A, S5A, S6A). In terms of trends, higher species richness (Chao1) was observed in the microbial community within samples from the “Production” and “Packaging Room B” compared to “Packaging Room A”, as well as in the microbiota within *Listeria* positive samples compared to *Listeria* negative samples. The alpha indices Observed (*p* = 0.02), Chao1 (*p* = 0.018), ACE (*p* = 0.025) and Fisher (*p* = 0.02) were significantly higher within samples from steel surfaces compared to plastic surfaces indicating higher species richness on steel surfaces (Supplementary Figure S7A).

A PERMANOVA analysis was conducted to statistically assess beta diversity among the different sampling groups. Microbial communities were significantly different (*p* = 0.034) between *Listeria*-positive and *Listeria*-negative samples (Supplementary Figure S4B). In addition, the different room types (“Production,” “Packaging Room A,” “Packaging Room B”; *p* = 0.0001) and the surface material (“steel,” plastic”; *p* = 0.021) were significant factors influencing the microbial community structure (Supplementary Figures S6B, S7B). No significant differences between the microbiota in the biofilm-positive and -negative sites were detected (Supplementary Figure S5B, *p* = 0.262).

**Figure 3:**
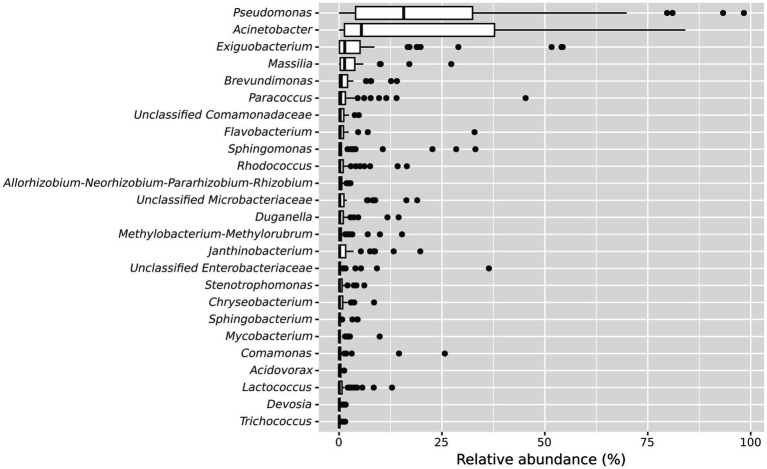
Boxplot showing the 25 most abundant genera within all samples from the frozen vegetable processing environment. Genera are decreasingly ordered by their relative median abundance.

Further, we examined the taxonomic composition of the microbial community in the frozen vegetable processing environment. The 10 most abundant genera in microbiota of the processing facility across all samples were *Pseudomonas* (median relative abundance: 15.7%), *Acinetobacter* (median relative abundance: 5.43%), *Exiguobacterium* (median relative abundance: 1.39%), *Massilia* (median relative abundance: 1.35%), *Brevundimonas* (median relative abundance: 0.56%), *Paracoccus* (median relative abundance: 0.39%), unclassified *Comamonadaceae* (median relative abundance: 0.28%), *Flavobacterium* (median relative abundance: 0.27%), *Sphingomonas* (median relative abundance: 0.22%) and *Rhodococcus* (median relative abundance: 0.21%) (Figure 3).

**Figure 4:**
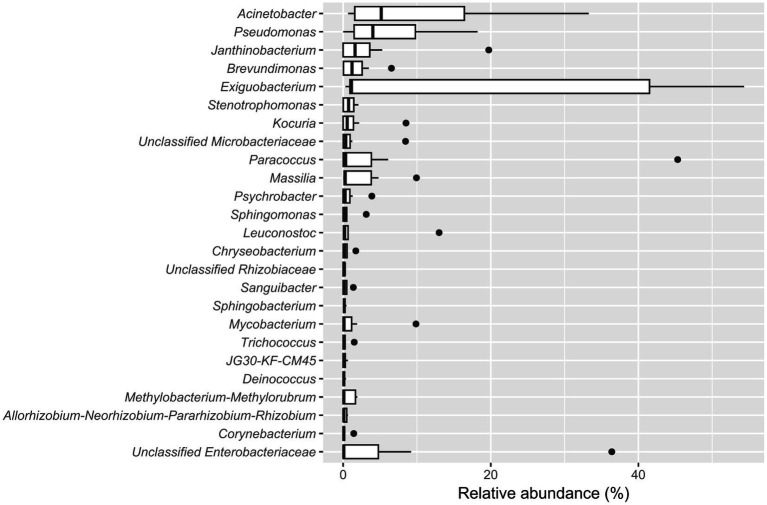
Boxplot showing the 25 most abundant genera within samples from the biofilm-positive sample group. Genera are decreasingly ordered by their relative median abundance.

The five most abundant genera in the biofilm-positive sample group were *Acinetobacter* (median relative abundance: 5.17%), *Pseudomonas* (median relative abundance: 4.02%), *Janthinobacterium* (median relative abundance: 1.61%), *Brevundimonas* (median relative abundance: 1.20%), and *Exiguobacterium* (median relative abundance: 1.13%) (Figure 4).

#### 3.4.1 Differential abundance of ASVs in *Listeria*-positive versus *Listeria*-negative samples

**Figure 5:**
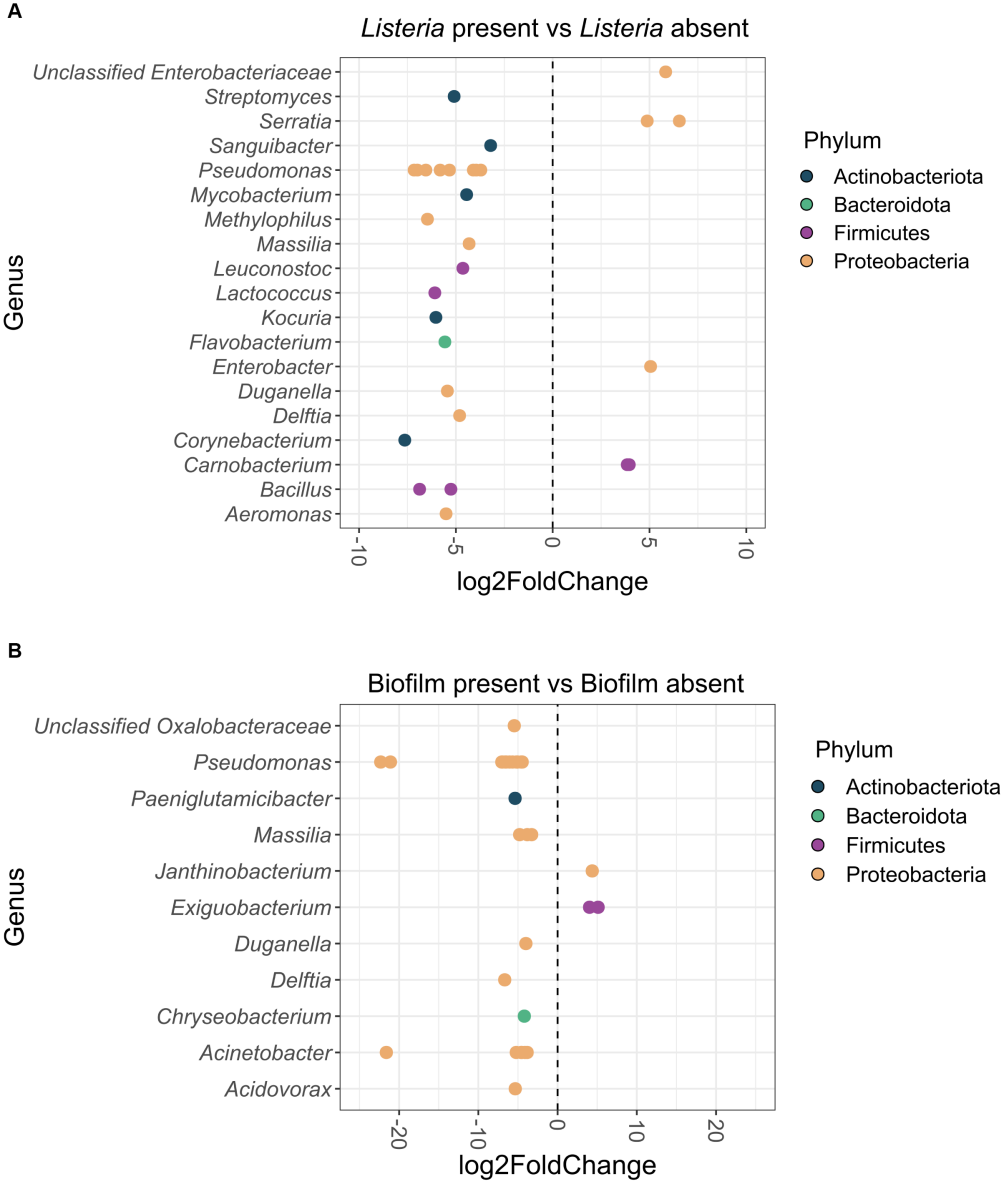
**(A)** Differentially abundant (*p* < 0.05) ASVs at genus-level in the *Listeria*-positive versus *Listeria*-negative sample group. Positive log_2_Fold Change values indicate a higher abundance of ASVs in the *Listeria*-positive sample group and negative log_2_Fold Change values indicate a lower abundance of ASVs in the *Listeria*-positive sample group. ASVs are displayed as dots for each separate genus. ASVs are additionally color-coded by phylum. **(B)** Differentially abundant (*p* < 0.05) ASVs at genus-level from biofilm-positive versus biofilm-negative sites. Positive log_2_Fold Change values indicate a higher abundance of ASVs in the biofilm-positive sample group and negative log_2_Fold Change values indicate a lower abundance of ASVs in the biofilm-positive sample group. ASVs are displayed as dots for each separate genus. ASVs are additionally color-coded by phylum.

In total, 30 ASVs were significantly differentially abundant between *Listeria*-positive and -negative samples (Figure 5A). Of these, six ASVs classified as *Carnobacterium*, *Enterobacter*, *Serratia* and unclassified *Enterobacteriaceae* were more abundant when *Listeria* was present. The other 24 differentially abundant ASVs, assigned to 15 different genera, were significantly more abundant in *Listeria*-negative samples (*Aeromonas*, *Bacillus*, *Corynebacterium*, *Delftia*, *Duganella*, *Flavobacterium*, *Kocuria*, *Lactococcus*, *Leuconostoc*, *Massilia*, *Methylophilus*, *Mycobacterium*, *Pseudomonas*, *Sanguibacter*, *Streptomyces*).

#### 3.4.2 Differential abundance of ASVs in samples from biofilm harboring sites

When comparing biofilm-positive and -negative sites, 31 ASVs were significantly differentially abundant (Figure 5B). Three ASVs classified as *Exiguobacterium* and *Janthinobacterium* were more abundant in biofilms, whereas 28 ASVs classified as *Acidovorax*, *Acinetobacter*, *Chryseobacterium*, *Delftia*, *Duganella*, *Massilia*, *Paeniglutamicibacter*, *Pseudomonas*, and unclassified *Oxalobacteraceae* were significantly more abundant on biofilm-negative sites.

#### 3.4.3 Differential abundance of ASVs in different rooms of the frozen vegetable processing facility

Differential abundance analysis identified 126 ASVs from 60 different genera that were significantly differentially abundant between “Packaging Room A” and the “Production” room (Supplementary Figure S8), and 79 ASVs from 43 genera between “Packaging Room B” and the “Production” room (Supplementary Figure S9). Only 34 ASVs of 12 genera were differentially abundant between “Packaging Room A” and “Packaging Room B” (Supplementary Figure S10).

In “Packaging Room A”, 23 ASVs, among them prevalent genera such as *Exiguobacterium*, *Pseudomonas*, *Acinetobacter*, and *Psychrobacter*, were significantly more abundant than in the “Production” room. Also, 123 ASVs, such as those assigned to *Pseudomonas*, *Exiguobacterium*, *Acinetobacter*, or *Massilia*, were significantly more abundant in the “Production” room compared to “Packaging Room A.

In “Packaging Room B”, 14 ASVs were significantly more abundant compared to the “Production” room, among them genera such as *Pseudomonas*, *Exiguobacterium* or unclassified *Enterobacteriaceae*. Other ASVs of prevalent genera such as *Acinetobacter*, *Exiguobacterium*, *Massilia, Paracoccus* and *Pseudomonas* were significantly more abundant in the “Production” room than in “Packaging Room B.” The majority of differentially abundant ASVs (65 ASVs) were significantly more abundant in “Production” when compared to “Packaging Room B”.

In “Packaging Room A”, 22 ASVs were significantly more abundant than in “Packaging Room B,” among them ASVs assigned to *Acinetobacter*, *Corynebacterium*, *Exiguobacterium*, *Massilia*, *Pseudomonas*, *Psychrobacter*, and *Sphingobacterium*, and 12 ASVs (*Brevundimonas*, *Enterobacter*, *Exiguobacterium*, *Kocuria*, *Pseudomonas*, *Sphingomonas*, and unclassified *Enterobacteriaceae*) were significantly lower abundant.

#### 3.4.4 Differential abundance of ASVs between steel and plastic surfaces

Differential abundance of ASVs between samples from steel and plastic surfaces was additionally assessed. 51.61% of samples from the packaging rooms and 67.5% from the “Production” room collected from steel surfaces. We identified 104 ASVs assigned to 52 genera that were significantly differentially abundant between samples from steel versus plastic surfaces (Supplementary Figure S11). In samples from steel surfaces, 86 ASVs were identified as significantly more abundant, among them were 27 ASVs of prevalent genera such as *Acinetobacter*, *Enterococcus*, *Exiguobacterium*, *Flavobacterium*, *Massilia*, *Paracoccus*, *Pseudomonas*, and *Sphingomonas*. Of note, a similar number of differentially abundant ASVs were identified when both packaging rooms were compared to the “Production” room.

## 4 Discussion

This study aimed to investigate the presence of *Listeria* in biofilms and characterize the co-existing microbiota in the processing environment of a European frozen vegetable processing facility, where *L. monocytogenes* in-house clones persisted for years (Pracser et al., 2024). We collected all samples after cleaning and disinfection to assess the hygiene status of the processing environment while avoiding the presence of food residues, which would interfere with the analysis of extracellular matrix components of biofilms.

The microbiota in the processing facility, where bacteria encounter temperatures ranging from −20°C to +70°C and significant moisture, was dominated by *Pseudomonas*, *Acinetobacter* and *Exiguobacterium*. While *Exiguobacterium* is often found in extreme environments (Vishnivetskaya et al., 2009), *Pseudomonas* and *Acinetobacter* are commonly found in diverse food processing environments (Zwirzitz et al., 2021; Rolon et al., 2023; Serrano Heredia et al., 2023; Tadielo et al., 2023; Valentino et al., 2023; Xu et al., 2023). In addition, members of *Pseudomonas* (Zhang et al., 2019; Chauhan et al., 2023), *Acinetobacter* (Kim et al., 2018; Herrera et al., 2021) and *Exiguobacterium* (Vishnivetskaya et al., 2007; Rodrigues et al., 2008) are capable of adapting to low temperatures.

In this study, biofilms were detected on 12.7% (9/71) of the sampled sites, which is in line with other studies using similar methods for biofilm detection. Previous research reported a biofilm prevalence of 9.3% in a meat processing environment (Wagner et al., 2020) and 17% across multiple types of food processing environments, including those producing oven foods, dairy products, meat products, baker’s yeast, sauces and egg products (Maes et al., 2017). In our study, biofilms were mainly located on direct and indirect food contact surfaces such as on conveyor belts and conveyor belt guide rollers (44% of biofilm positive sites), a screw conveyor, a metal tub, an air separator, and two steel funnels. Moreover, the highest bacterial loads were found in biofilms located on a screw conveyor (5.8 log BCE/cm^2^) and two conveyor belt guide rollers (5.4 log BCE/cm^2^, 4.9 log BCE/cm^2^). It is well known that biofilms have high cell densities (Flemming et al., 2016). On sites with attached bacterial cells and established biofilms, chemical cleaning together with mechanical cleaning strategies such as scraping or enzymatic disruption of biofilms would be essential for biofilm removal (Chmielewski and Frank, 2003; Kumar et al., 2021; Mazaheri et al., 2022). The top five most abundant genera within the biofilm-positive sample group were *Acinetobacter*, *Pseudomonas*, *Janthinobacterium*, *Brevundimonas*, and *Exiguobacterium*. Different studies have demonstrated the biofilm-forming ability of all five of these genera (Pantanella et al., 2007; Douterelo et al., 2014; Chauhan et al., 2018; Wagner et al., 2021; Chernogor et al., 2022; Gricajeva et al., 2022; Huang et al., 2022; Liu et al., 2023; Maifreni et al., 2023). In addition, *Acinetobacter*, *Pseudomonas*, *Janthinobacterium*, and *Brevundimonas* were previously found in biofilms in food processing environments (Dzieciol et al., 2016; Wagner et al., 2020). In biofilms, *Janthinobacterium* and *Exiguobacterium* were more abundant than in the other samples. Some *Exiguobacterium* species have been described as having the potential to degrade plastic materials such as polystyrene or polypropylene. The biodegrading activity of bacteria can lead to alterations in surface material properties, such as changes in hydrophobicity or damage to the material (Chauhan et al., 2018; Sun et al., 2023). Surface defects or increased roughness make surfaces challenging to clean, which is favorable for biofilm formation, as previously discussed (Dass and Wang, 2022; Sharan et al., 2022). In this study, 38.4% of sample surfaces were built from plastics. Although *Pseudomonas* was present in biofilm samples, we detected 13 significantly lower abundant *Pseudomonas* ASVs in biofilms compared to biofilm-negative sites. This was unexpected, as *Pseudomonas* is a frequent biofilm-former and *Pseudomonas* biofilms were described to provide other bacterial species, e.g., *L. monocytogenes*, protection from environmental stressors such as cleaning and disinfection (Sterniša et al., 2023; Thomassen et al., 2023; Zarei et al., 2023). Since *Pseudomonas* is widespread in the food processing environment, we assume that species or strain-level differences are responsible for the findings of our study. This hypothesis is supported by a study which demonstrated inter- and intra-species differences in the biofilm-forming ability of *Pseudomonas* isolates from meat and dairy processing environments (Fernández-Gómez et al., 2021). Microbial interactions may pose another factor influencing the microbiota at biofilm-positive sites, since Singh et al. (2019) reported an anti-biofilm activity of *Exiguobacterium indicum* against *Pseudomonas aeruginosa* by quorum sensing inhibition. Nevertheless, further research with additional data, such as metagenomes or from the characterization of isolates would be required to explore functional correlations at the species and strain levels.

Different studies hypothesized that (multispecies) biofilms support *Listeria* persistence in food processing environments, as the biofilm matrix protects *Listeria* from disinfectants (Van der Veen and Abee, 2011; Oxaran et al., 2018; Rolon et al., 2024b). In this study, we detected *L. innocua* on two biofilm harboring sites, located at conveyor belt guide rollers with *Exiguobacterium* present in abundance. These results demonstrate for the first time that *Listeria* spp. are able to colonize biofilms in the processing environment. Due to the low sample number, further research would be necessary to test if *Exiguobacterium* supports *Listeria* survival and colonization (on plastic surfaces) under conditions found in food processing environments. Yet, no biofilm was detected on the majority of *Listeria*-positive sites (12/14 sites), indicating that biofilms did not play a significant role in *Listeria* survival and persistence. Both, *L. innocua* and *L. monocytogenes* were detected on conveyor belts and the associated conveyor belt guide rollers, and in drains. In previous studies, conveyor belts, especially modular-built conveyor belts with multiple joints, were described as hard-to-clean niches, which contribute to the survival of *Listeria* and the establishment of biofilms (Veluz et al., 2012; Langsrud et al., 2016; Fagerlund et al., 2017; Belias et al., 2022). The proportion of modular conveyor belts was higher in the packaging rooms than in the production room, which could explain the higher prevalence of biofilms and *Listeria* in the packaging rooms. In addition, differences in cleaning and disinfection efficiency (Burnett et al., 2022; Maillard and Centeleghe, 2023) or surface defects (Hua and Zhu, 2024) can also influence biofilm establishment and the survival of *Listeria*. It is frequently reported that drains serve as reservoirs for *Listeria* (Rückerl et al., 2014; Kaszoni-Rückerl et al., 2020; Stessl et al., 2020; Bardsley et al., 2024) and biofilms (Dzieciol et al., 2016; Wagner et al., 2020). In our study, we detected *Listeria* in five drains, but none of the total seven drains harbored a biofilm. Clearly, conveyor belts and drains provide a potential reservoir for *Listeria*. The difficulty to clean the type of modular conveyor belts used in the processing facility promotes the survival of *Listeria*. In floor drains, accumulation of organic matter, moisture and the potential presence of biofilms may provide beneficial conditions for *Listeria*.

Characterization of *Listeria* spp. revealed the presence of ST8 and ST224 *L. monocytogenes* isolates in the processing environment. Both MLST-STs were prevalent in the same frozen vegetable processing environment in our previous study. ST224-CT5623 was identified as an in-house clone in the packaging area, specifically in “Packaging Room B” (Pracser et al., 2024). Our findings, combined with the results of our previous research, clearly show that ST224-CT5623 is still present after multiple cleaning and disinfection cycles. Furthermore, whole genome SNP analysis of *L. innocua* isolates in the ST1481 and ST2347 groups detected 0 and 0–2 SNPs, respectively, and isolates within a MLST-ST group clustered together with 100% bootstrap support in a phylogenetic tree, may indicating a close genetic relatedness in relation to the metadata (Jagadeesan et al., 2019).

We additionally investigated the microbiota co-occurring with *Listeria* in the frozen vegetable processing environment. In different food producing environments, different genera have been found to correlate with the presence of *Listeria*. For example, *Pseudomonas*, *Acinetobacter*, or *Janthinobacterium* were reported to be indicative for *Listeria* presence in a meat processing environment (Zwirzitz et al., 2021). Also, *Pseudomonas*, *Stenotrophomonas*, and *Microbacterium* were associated with *Listeria* presence in tree fruit packing facilities (Rolon et al., 2024b), and *Carnobacterium*, *Psychrobacter*, and *Pseudomonas* were more abundant in samples positive for *Listeria* in distribution centers for fresh produce (Townsend et al., 2023). We also detected significant differences in the community structure between *Listeria*-positive and *Listeria*-negative samples. *Carnobacterium*, *Enterobacter*, *Serratia* and ASVs from unclassified *Enterobacteriaceae* were found to be higher abundant in *Listeria*-positive samples. Interestingly, several *Pseudomonas* ASVs were less abundant in *Listeria*-positive compared to *Listeria*-negative samples. This is in line with another study conducted in a meat processing environment, which reported a higher relative abundance of *Pseudomonas* (not significant) in *Listeria*-negative samples (Belk et al., 2022), but is inconsistent with the results from Zwirzitz et al. (2021), where the presence of *Listeria* was associated with the occurrence of *Pseudomonas*. To further analyse this discrepancy, we compared the significantly lower abundant *Pseudomonas* ASVs (from partial 16S rRNA gene sequencing) in *Listeria*-positive samples of this study, to the significantly higher abundant *Pseudomonas* ASVs (nearly full length 16S rRNA gene sequencing) in *Listeria*-positive samples of the study from Zwirzitz et al. (2021). Our analysis detected 100% nucleotide identity (compared to the sequences from Zwirzitz et al., 2021) for only one *Pseudomonas* ASV, whereas all other ASVs shared between 95.79 and 99.07% nucleotide identity. This finding indicates that species- and/or strain-level differences are among the driving forces for shaping the microbial community. Therefore, further research using whole metagenome sequencing rather than 16S rRNA gene sequencing is necessary to investigate the microbial community structure on species and strain level.

Similar to the findings of Belk et al. (2022) and other studies (Dzieciol et al., 2016; Cobo-Díaz et al., 2021), we found distinct specific communities in various areas of food processing environments. Major differences were observed between the “Production” room and both packaging rooms, as in the “Production” room 123 ASVs were significantly more abundant than in “Packaging Room A” and 65 ASVs compared to “Packaging Room B”. The variance in the composition of the microbiota may be explained by environmental conditions such as temperature. In the packaging rooms, the temperature ranged from cold to ambient conditions, whereas in the “Production” room mostly ambient to hot conditions were predominant during operation. Furthermore, we observed significant variations in alpha-diversity measures (Observed, Chao1, ACE, Fisher) and in beta-diversity when we compared the microbiota of samples from steel and plastic surfaces. Surprisingly, in samples from steel surfaces, we found higher richness within the microbiota. Differential abundance analysis revealed 86 ASVs were higher in abundance in samples from steel surfaces than in samples from plastic surfaces, mainly built from polyacetal. The materials have different surface properties, as for example steel is generally more hydrophilic, whereas polyacetal is rather hydrophobic. There is evidence that bacterial attachment to surfaces is influenced by individual strain abilities, roughness, and hydrophobicity of the surface material (Ashok et al., 2023; Cai et al., 2023; Finn et al., 2023), which may explain the variations in the microbiota of steel and plastic surfaces. For instance, significantly more biomass of *Acinetobacter baumannii* accumulated on polycarbonate compared to other surface materials such as glass, rubber, porcelain and polypropylene in a study (Greene et al., 2016). An increased albeit not significant biofilm formation of *Pseudomonas fluorescens* was observed on polystyrene compared to glass and stainless steel (Gagné et al., 2022). However, the factors influencing attachment and biofilm formation on different surface materials are complex. A study reported a significantly increased biofilm formation of *Pseudomonas fluorescens* on stainless steel at 25°C compared to 4°C. This temperature-dependent effect on biofilm formation was reversed but not significant on polystyrene and not visible on polytetrafluoroethylene (Maifreni et al., 2023). Moreover, the number of hard-to-clean niches in the two sample groups and/or the sanitation efficacy on the plastic and steel surface materials may affect the composition of the microbiota, as a study reported differences in cleaning and disinfection efficacy of smooth plastic surfaces built from high-density polyethylene surfaces compared to stainless-steel (Ohman et al., 2023). The frozen vegetable producer applies multiple cleaning and disinfection agents including chlorine-based agents, foaming surfactants and peracetic acid. There were no differences in the cleaning and disinfection regime between the different rooms. There is still a gap in knowledge about which factors are the (main) drivers for shaping the microbiota. It is unclear if the differences in the microbiota were driven by the surface material or by environmental conditions in the rooms. The fact that different *Pseudomonas* ASVs were either higher or lower abundant in samples from steel surfaces further supports potential differences at the species or strain level and their colonization of niches with distinct properties in food processing environments.

In summary, we detected *Listeria* spp. on 14 sites after cleaning and disinfection in the frozen vegetable processing environment. Of note is that the quality control team of the frozen vegetable processing facility regularly monitors the microbial safety of food products, which strongly reduces the risk for customers. *Listeria* spp. were present in two out of nine biofilms, demonstrating (to our knowledge) for the first time that *Listeria* spp. are a member of the microbial community in “real” biofilms in a food processing environment. Biofilms were detected by determining the presence of bacteria by qPCR and the presence of at least two extracellular matrix components of the biofilm (carbohydrates, proteins, eDNA). The finding that the majority of *Listeria* were found on non-biofilm sites indicates that biofilms are only one factor for *Listeria* survival in the processing environment. Biofilms and *Listeria* were frequently found on modular conveyor belts and/or conveyor belt guide rollers, hard-to-clean niches which showed the highest bacterial load. Therefore, we recommend substituting these conveyors to enhance hygiene in the frozen vegetable processing facility. ASVs assigned to *Carnobacterium*, *Serratia*, *Enterobacter* and unclassified *Enterobacteriaceae* were associated with *Listeria* presence. *Janthinobacterium* and *Exiguobacterium*, both known biofilm formers, were significantly more abundant in biofilms. In addition, there were indications that the different room types and their specific environmental factors, such as temperature or surface materials, play a role in shaping the existing microbiota. Our findings demonstrate the complex nature of microbial interactions in the food-producing environment, including biofilm and other *Listeria* niches, and suggest that further research is needed to identify the main drivers for modulating the microbiota and the presence of *Listeria* in food-processing environments.

## Data availability statement

The datasets presented in this study can be found in online repositories. The datasets analyzed for this study can be found in the NCBI repository under the BioProject number PRJNA1075930 https://www.ncbi.nlm.nih.gov/.

## Author contributions

NP: Conceptualization, Data curation, Formal analysis, Investigation, Methodology, Visualization, Writing – original draft, Writing – review & editing. EV: Conceptualization, Methodology, Project administration, Supervision, Writing – review & editing. ST: Data curation, Investigation, Methodology, Writing – review & editing. AP: Data curation, Methodology, Writing – review & editing, Investigation. ES: Conceptualization, Formal analysis, Supervision, Writing – review & editing. MW: Funding acquisition, Resources, Supervision, Writing – review & editing, Project administration. KR: Conceptualization, Formal analysis, Funding acquisition, Project administration, Supervision, Writing – original draft, Writing – review & editing.
