# Supplementary material for: Exploring the occurrence of Listeria in biofilms and deciphering the bacterial community in a frozen vegetable producing environment
Source: Front Microbiol. 2024 Jul 10;15:1404002. doi: 10.3389/fmicb.2024.1404002 (PMC11266072; doi:10.3389/fmicb.2024.1404002)
Supplement: Supplementary file 1 [file Data_Sheet_1.PDF]

Supplementary Material

1 List of supplementary figures

Fig S1: EPS matrix content

Fig S2: Maximum likelihood phylogeny based on whole genome SNP data (*L. innocua*)

FigS3: Maximum likelihood phylogeny based on whole genome SNP data (*L. monocytogenes*)

Fig S3: Alpha diversity indices and clustering of *Listeria* positive and *Listeria* negative sample groups using Bray-Curtis dissimilarity

Fig S3: Alpha diversity indices and clustering of biofilm positive and biofilm negative sample groups using Bray-Curtis dissimilarity

Fig S4: Alpha diversity indices and clustering of different room type groups ("Production", "Packaging Room A", "Packaging Room B") using Bray-Curtis dissimilarity

Fig S5: Alpha diversity indices and clustering of surface type sample groups (steel vs plastic) using Bray-Curtis dissimilarity

Fig S6: Differential abundance analysis of ASVs between "Packaging Room A" and the "Production" room

Fig S7: Differential abundance analysis of ASVs between "Packaging Room B" and the "Production" room

Fig S8: Differential abundance analysis of ASVs between "Packaging Room A" and the "Packaging Room B" room

Fig S9: Differential abundance analysis of ASVs between samples collected from steel and plastic surfaces

2 Supplementary Figures

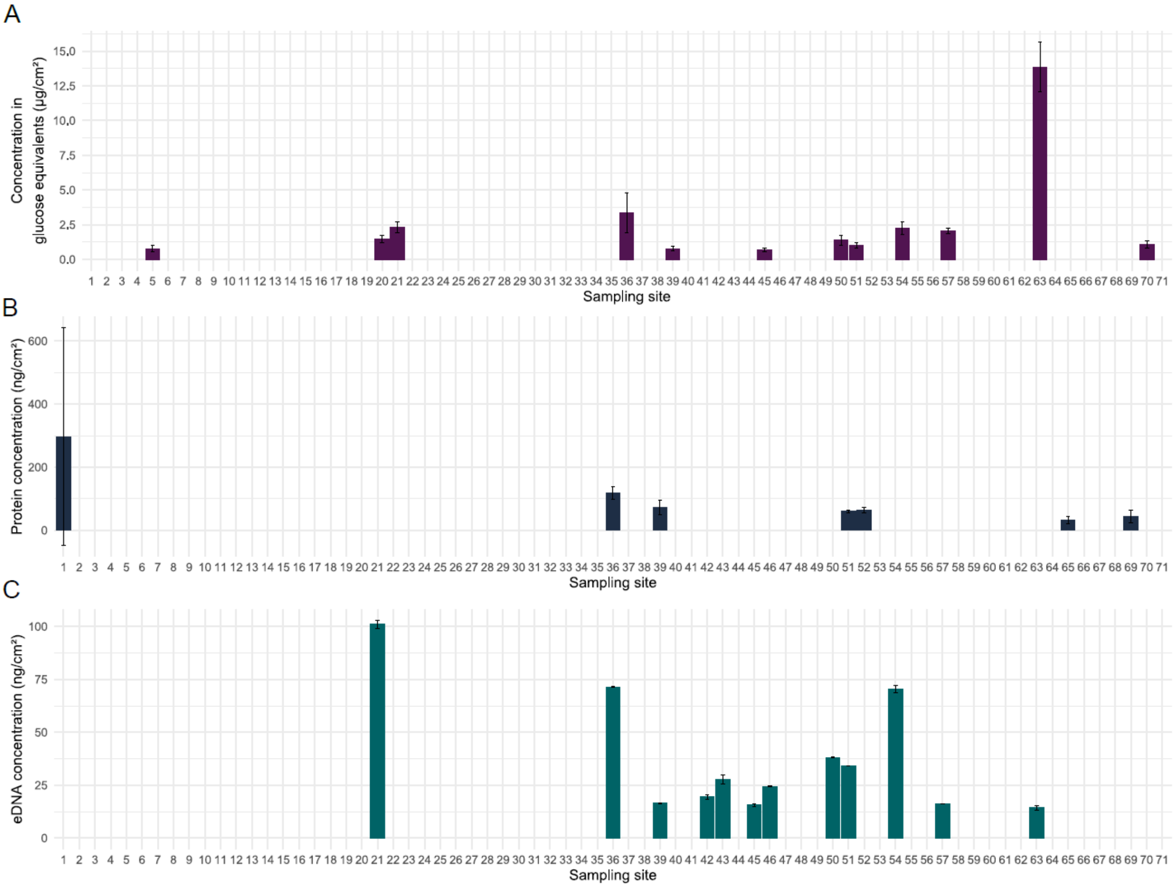

Figure S1 EPS matrix content in samples collected with scraper and flocked swabs. **(A)** Glucose equivalents in µg/cm² (mean ± SD) per sample. **(B)** Protein content in ng/cm² (mean ± SD) **(C)** eDNA concentration in ng/cm² (mean ± SD).

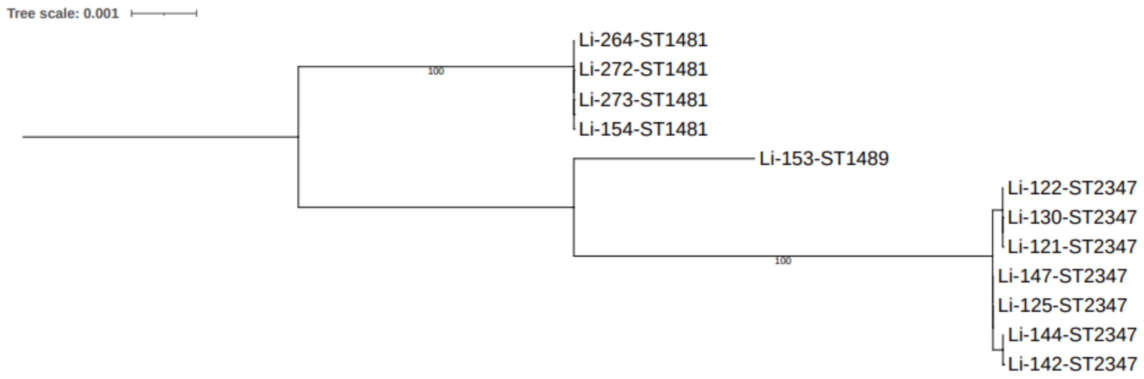

Figure S2 Maximum likelihood phylogeny (midpoint rooted) based on whole genome SNP data showing *L. innocua* isolates from the frozen vegetable processing environment.

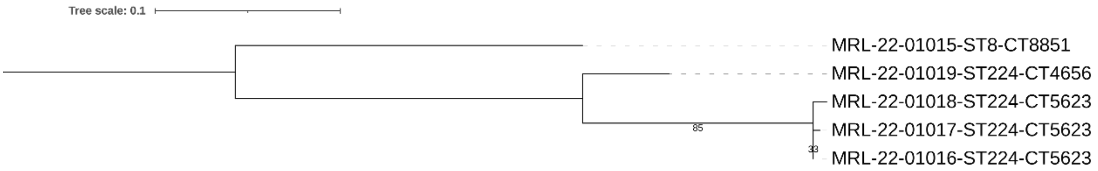

Figure S3 Maximum likelihood phylogeny (midpoint rooted) based on whole genome SNP data showing *L. monocytogenes* isolates from the frozen vegetable processing environment.

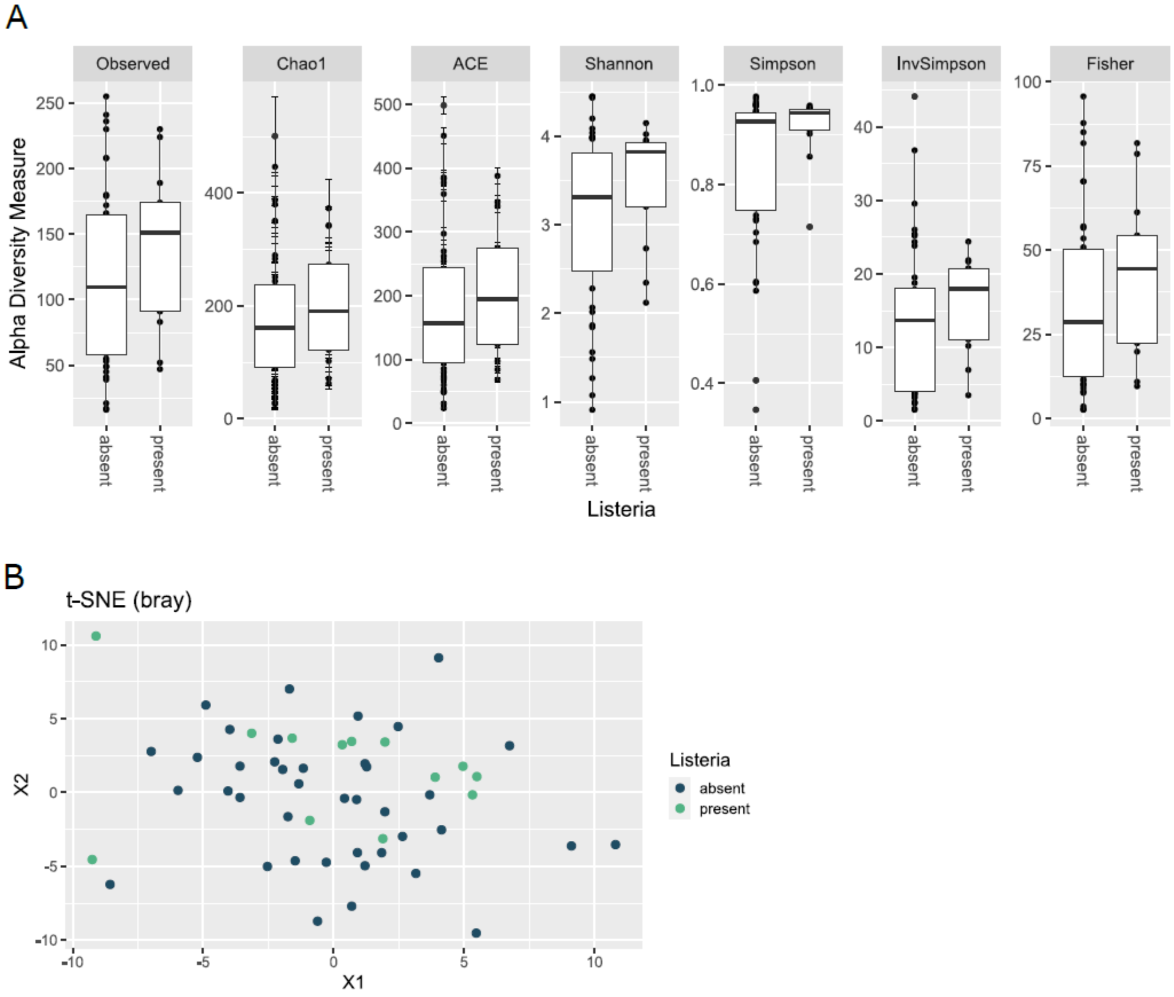

Figure S4 (A) Alpha diversity indices (Observed, Chao1, ACE, Shannon, Simpson, InvSimpson, Fisher) for the microbiome within the *Listeria* positive and *Listeria* negative sample groups. **(B)** Clustering of the *Listeria* present and *Listeria* absent sample groups by T-Distributed Stochastic Neighbor Embedding (t-SNE) plot using Bray-Curtis dissimilarity.

A

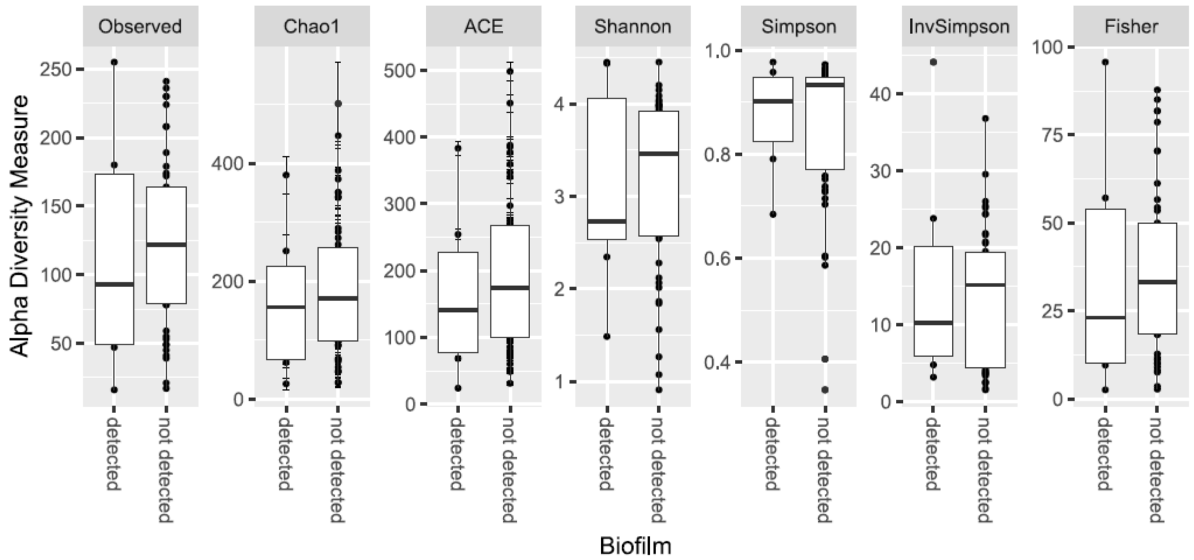

B

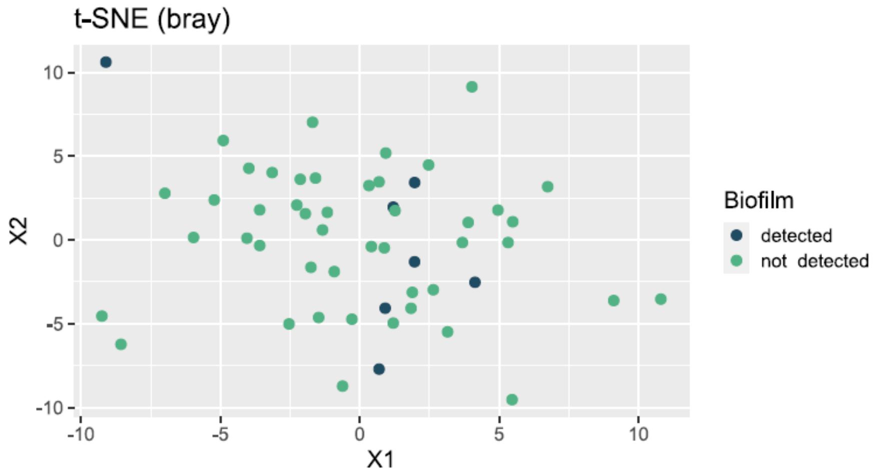

Figure S5 (A) Alpha diversity indices (Observed, Chao1, ACE, Shannon, Simpson, InvSimpson, Fisher) for the microbiome within biofilm positive and negative sample groups (biofilm not detected). **(B)** Clustering of biofilm positive and negative samples by T-Distributed Stochastic Neighbor Embedding (t-SNE) plot using Bray-Curtis dissimilarity.

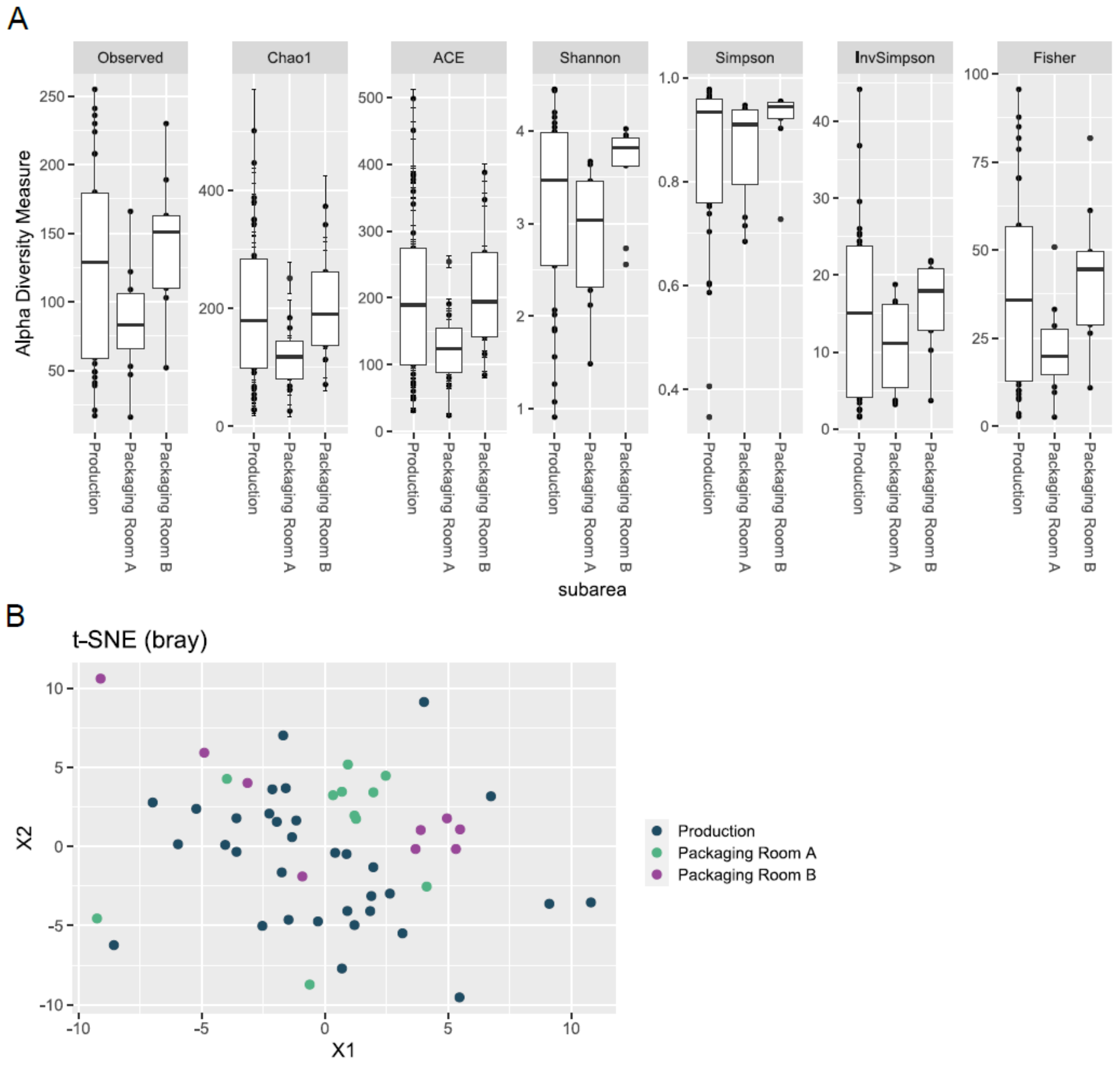

Figure S6 (A) Alpha diversity indices (Observed, Chao1, ACE, Shannon, Simpson, InvSimpson, Fisher) for the microbiome within sample groups collected in different rooms ("Production", "Packaging Room A", "Packaging Room B"). **(B)** Clustering of samples in different room categories by T-Distributed Stochastic Neighbor Embedding (t-SNE) plot using Bray-Curtis dissimilarity.

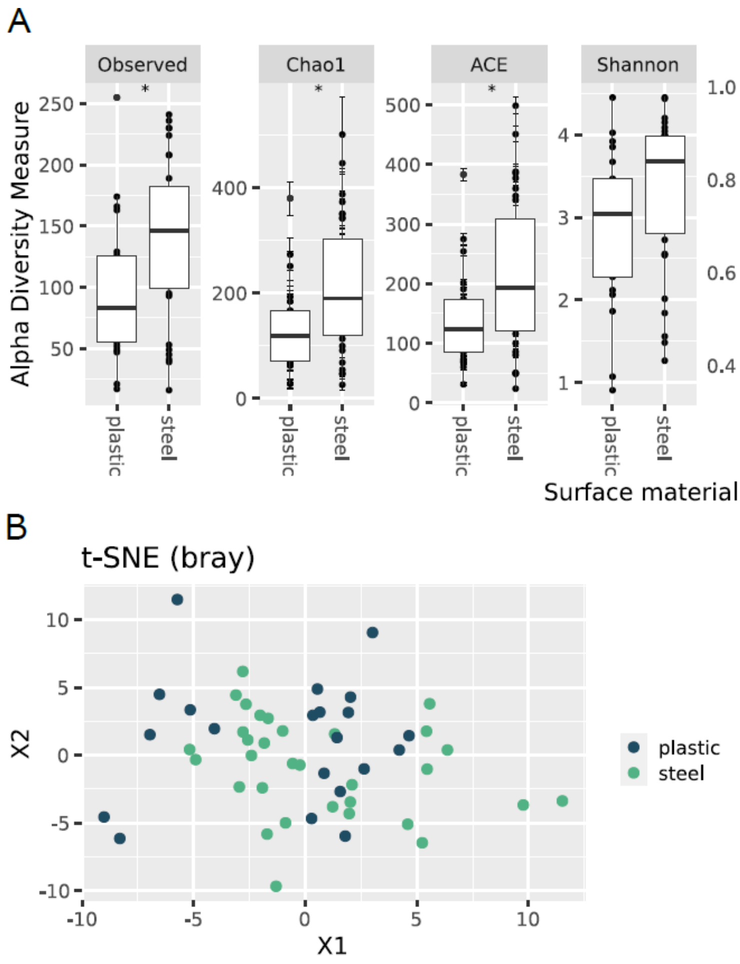

Figure S7 (A) Alpha diversity indices (Observed, Chao1, ACE, Shannon, Simpson, InvSimpson, Fisher) for the microbiome within sample groups collected from steel and plastic surfaces. **(B)** Clustering of samples from steel and plastic surfaces by T-Distributed Stochastic Neighbor Embedding (t-SNE) plot using Bray-Curtis dissimilarity.

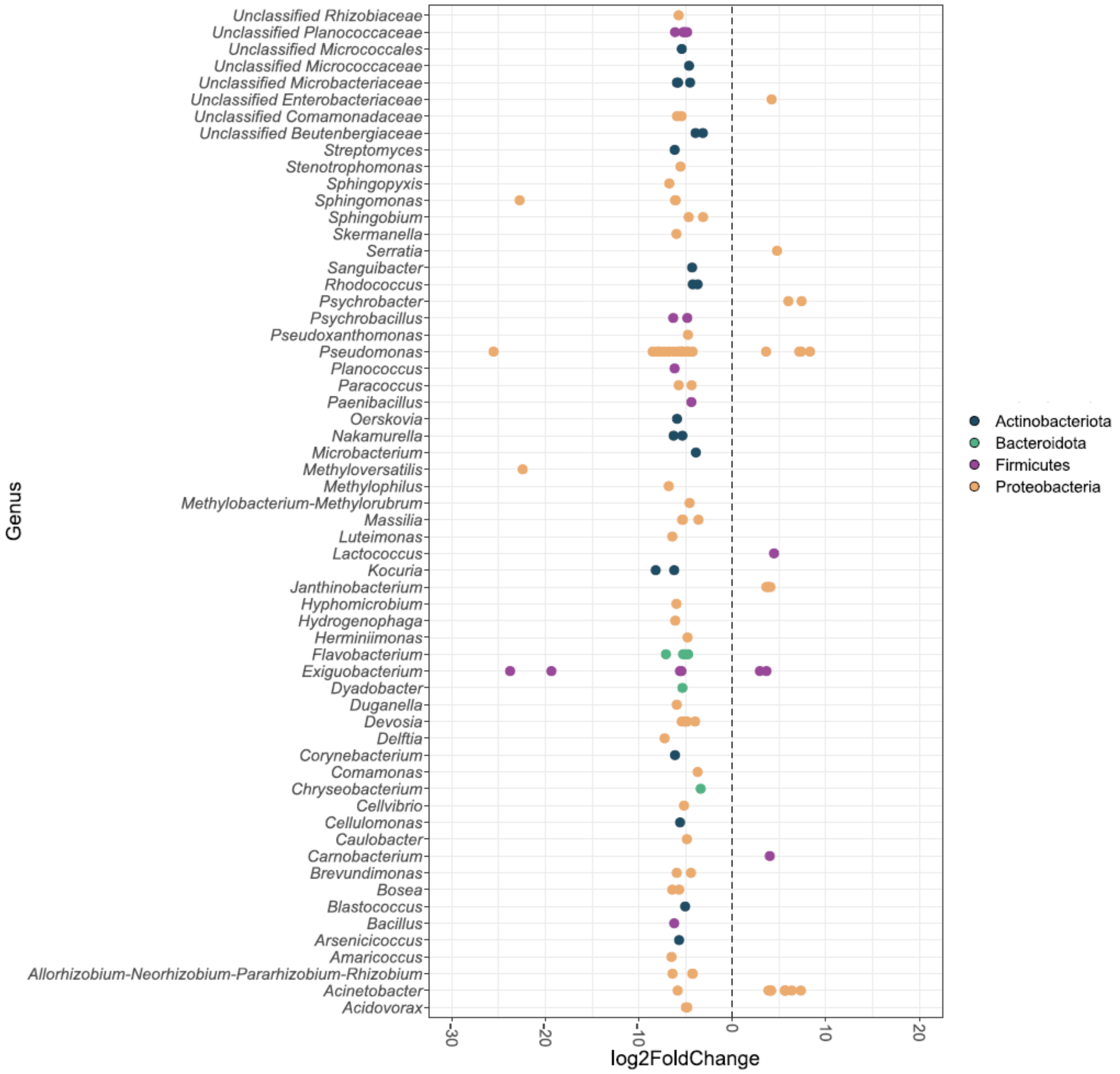

Figure S8 Significantly differentially abundant ASVs ($p < 0.05$) between "Packaging Room A" and the "Production" room. Positive log2FoldChange values indicate a higher abundance of ASVs in the "Packaging Room A" sample group and negative log2FoldChange values indicate a lower abundance of ASVs in the "Packaging Room A" sample group. ASVs are displayed as dots for each separate genus. ASVs are additionally color-coded by phylum.

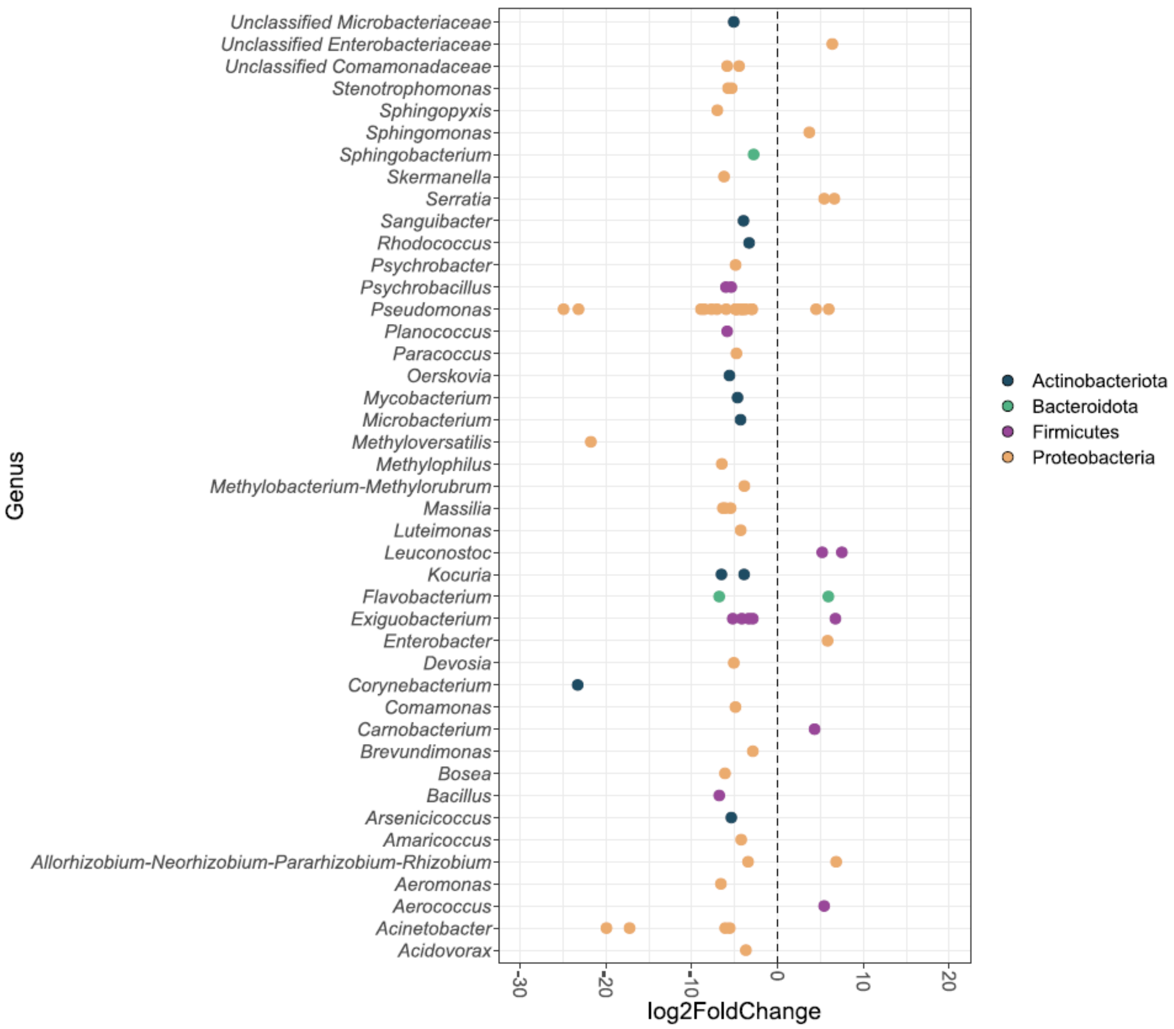

Figure S9 Significantly differentially abundant ASVs ($p < 0.05$) between “Packaging Room B” and the “Production” room. Positive log2FoldChange values indicate a higher abundance of ASVs in the “Packaging Room B” sample group and negative log2FoldChange values indicate a lower abundance of ASVs in the “Packaging Room B” sample group. ASVs are displayed as dots for each separate genus. ASVs are additionally color-coded by phylum.

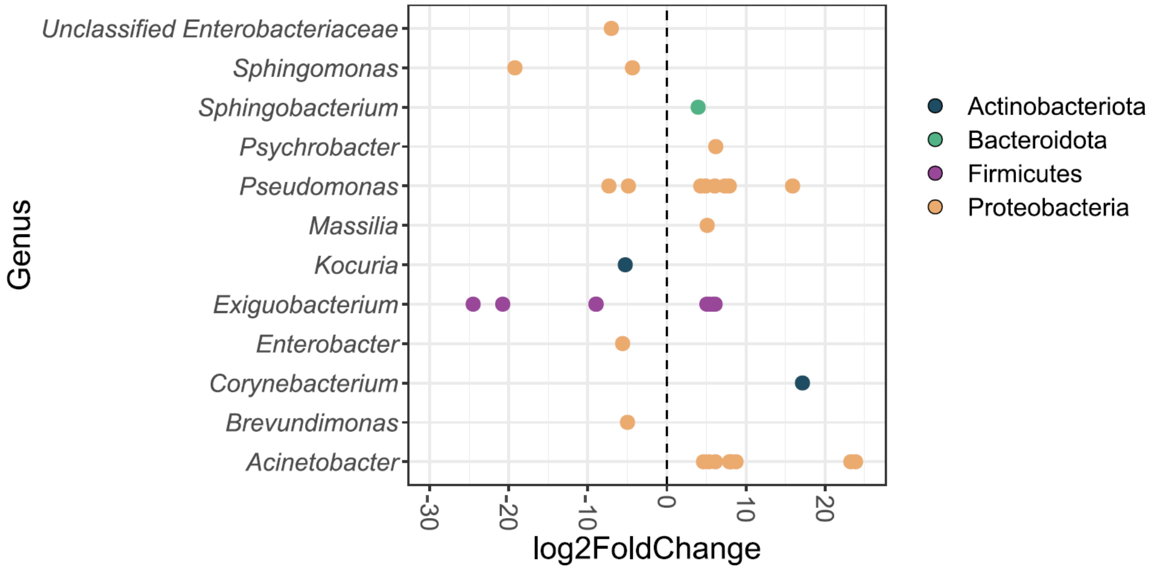

Figure S10 Significantly differentially abundant ASVs ($p < 0.05$) between “Packaging Room A” and the “Packaging Room B”. Positive log2FoldChange values indicate a higher abundance of ASVs in the “Packaging Room A” sample group and negative log2FoldChange values indicate a lower abundance of ASVs in the “Packaging Room A” sample group. ASVs are displayed as dots for each separate genus. ASVs are additionally color-coded by phylum.

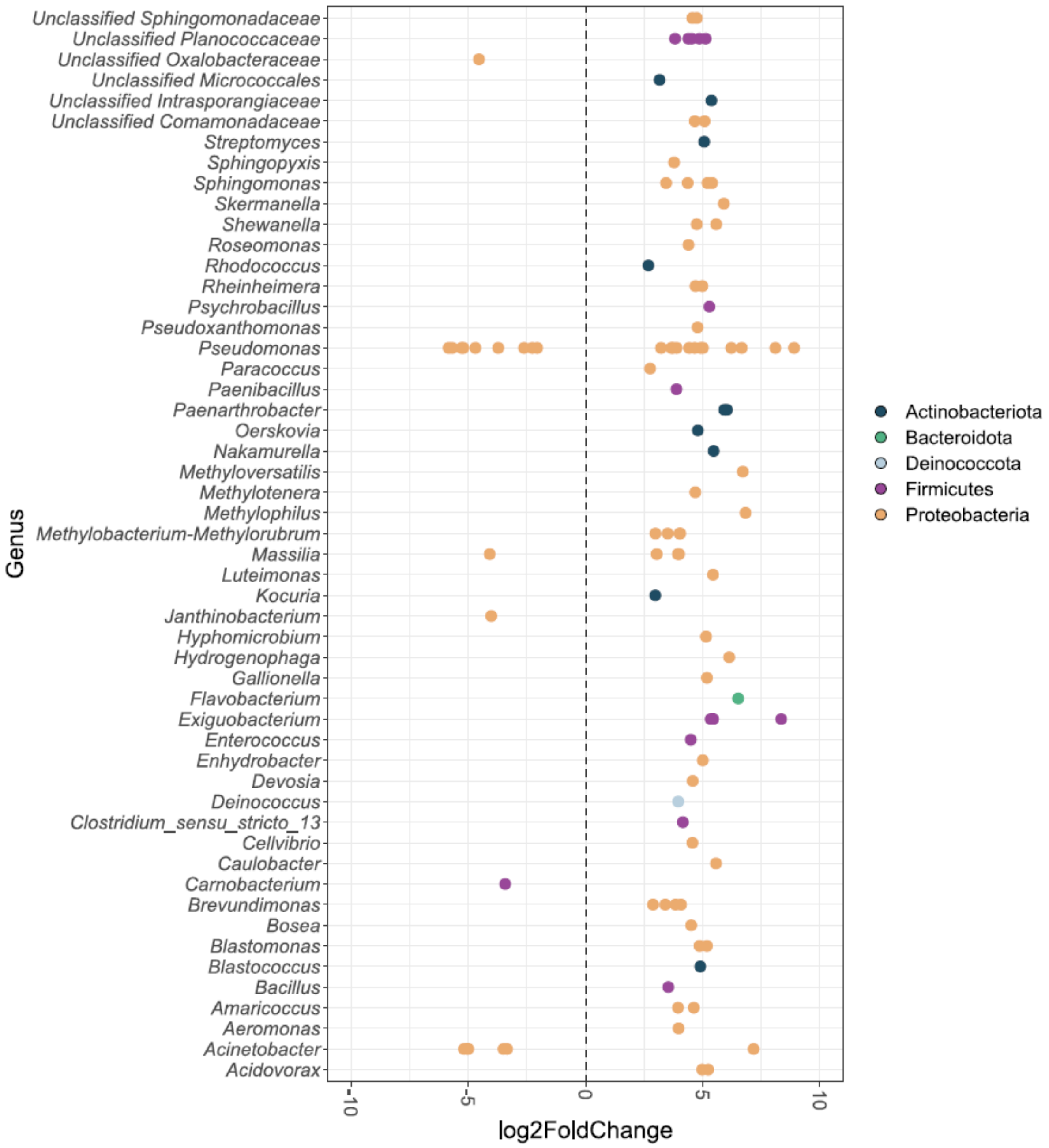

Figure S11 Significantly differentially abundant ASVs ($p < 0.05$) between samples grouped by surface material (steel vs plastic). Positive log2FoldChange values indicate a higher abundance of ASVs in the steel sample group and negative log2FoldChange values indicate a lower abundance of ASVs in the steel sample group. ASVs are displayed as dots for each separate genus. ASVs are additionally color-coded by phylum.

3 List of supplementary tables in separate Excel file

Table S1 List of sampling sites and overview about *Listeria* presence, presence of biofilm matrix components and total bacterial cell count.

Table S2 List of supplementary samples and overview about *Listeria* presence, presence of biofilm matrix components and total bacterial cell count.

Table S3 List of *Listeria* isolates and subtyping results.

Table S4 SNP matrix of a whole genome SNP analysis with *L. innocua* isolates of this study.

Table S5 SNP matrix of a whole genome SNP analysis with *L. monocytogenes* isolates of this study.

Table S3 List of primers used in this study.
